# Phylogenomic Insights into Distribution and Adaptation of Bdellovibrionota in Marine Waters

**DOI:** 10.3390/microorganisms9040757

**Published:** 2021-04-03

**Authors:** Qing-Mei Li, Ying-Li Zhou, Zhan-Fei Wei, Yong Wang

**Affiliations:** 1 Institute of Deep Sea Science and Engineering, Chinese Academy of Sciences, Sanya 572000, China; liqm@idsse.ac.cn (Q.-M.L.); zhouyl@idsse.ac.cn (Y.-L.Z.); weizf@idsse.ac.cn (Z.-F.W.); 2 University of Chinese Academy of Sciences, Beijing 100049, China

**Keywords:** bdellovibrionota, deep sea, bacterial predator, phylogeny, metabolism

## Abstract

Bdellovibrionota is composed of obligate predators that can consume some Gram-negative bacteria inhabiting various environments. However, whether genomic traits influence their distribution and marine adaptation remains to be answered. In this study, we performed phylogenomics and comparative genomics studies using 132 Bdellovibrionota genomes along with five metagenome-assembled genomes (MAGs) from deep sea zones. Four phylogenetic groups, Oligoflexia, Bdello-group1, Bdello-group2 and Bacteriovoracia, were revealed by constructing a phylogenetic tree, of which 53.84% of Bdello-group2 and 48.94% of Bacteriovoracia were derived from the ocean. Bacteriovoracia was more prevalent in deep sea zones, whereas Bdello-group2 was largely distributed in the epipelagic zone. Metabolic reconstruction indicated that genes involved in chemotaxis, flagellar (mobility), type II secretion system, ATP-binding cassette (ABC) transporters and penicillin-binding protein were necessary for the predatory lifestyle of Bdellovibrionota. Genes involved in glycerol metabolism, hydrogen peroxide (H_2_O_2_) degradation, cell wall recycling and peptide utilization were ubiquitously present in Bdellovibrionota genomes. Comparative genomics between marine and non-marine Bdellovibrionota demonstrated that betaine as an osmoprotectant is probably widely used by marine Bdellovibrionota, and all the marine genomes have a number of genes for adaptation to marine environments. The genes encoding chitinase and chitin-binding protein were identified for the first time in Oligoflexia, which implied that Oligoflexia may prey on a wider spectrum of microbes. This study expands our knowledge on adaption strategies of Bdellovibrionota inhabiting deep seas and the potential usage of Oligoflexia for biological control.

## 1. Introduction

Bdellovibrionota, well known as Bdellovibrio and like organisms (BALOs), are Gram-negative bacterial predators and are isolated from various environments. Bdellovibrionota are small vibrio-shaped bacteria with a polar flagellum [1]. In 1965, the first free-living BALO, *Bdellovibrio bacteriovorus*, was reported, which led to a discovery that predation process of Bdellovibrionota includes two steps, (1) attachment and degradation of prey cell wall with glycanases and (2) digestion of prey cellular components with exoenzyme [2]. Using *Escherichia coli* as a prey model, experimental results showed that Bdellovibrionota could penetrate the host cell and then transform into a bdelloplast before being released from the host cell [3]. In 1979, Bdellovibrionota was found to be an agent against pathogens [4], indicating the usage of Bdellovibrionota as a new approach to biocontrol that fights against bacterial infections, particularly when multidrug-resistant (MDR) bacteria are increasingly concerned globally [5]. MDR bacterial infections have been successfully treated with the assistance of bacterial predation by Bdellovibrionota [6]. The prey spectrum of Bdellovibrionota has recently expanded to Gram-positive pathogenic bacteria such as Enterococcus [7].

The current Bdellovibrionota phylum consists of several classes according to the Genome Taxonomy Database (GTDB, Release 89) [8], such as Bacteriovoracia, Bdellovibrionota, Oligoflexia and others without a taxonomic assignment. They are inhabitants that can be identified in various environments, such as soil, freshwater (river, lake and spring), ocean, wastewater treatment bioreactor and sewage [9,10,11,12]. In different ecological niches, Bdellovibrionota could live with a prey-dependent or prey-independent lifestyle [11]. This provides a clue to the metabolic flexibility of Bdellovibrionota, as well as to different predatory mechanisms when invading periplasmic space or attaching to the external surface of prey bacterial cell [1,13]. As bacterial predators distributed in a wide variety of niches with diversified predatory approaches, ecological roles of Bdellovibrionota were probably underestimated.

The ocean is probably the biggest reservoir of bacteria. So far the reported obligatory predator bacteria mainly include Micavibrio (a genus of α-proteobacteria) and five families belonging to δ-proteobacteria [14]. Proteobacteria is a super phylum that constitutes Gram-negative bacteria and dominates the microbial communities in dark deep-sea zones [15]. Experiments had exhibited that Halobacteriovorax, affiliated with one group of Bdellovibrionota, could predate deep-sea bacterium *Vibrio parahaemolyticus* (Gram-negative bacterium) [16]. In nutrient-poor deep sea, Gram-negative bacteria may meet difficulties in synthesis biopolymers of key cellular structural components, such as peptidoglycan. Therefore, Bdellovibrionota can probably prey on more Gram-negative bacteria, which likely occurs ubiquitously in deep-sea environments [17]. Under oligotrophic deep-sea environment, the competition for nutrient among microorganisms was constantly intensive [18]. The adaptation strategy of Bdellovibrionota in marine environment is still an unresolved issue.

In this study, available Bdellovibrionota genomes were collected for phylogenomic analysis along with five representative Bdellovibrionota genomes binned from the marine metagenomes. The environmental sources of the genomes indicated that two subclades (Bacteriovoracia and Bdello-group2) were mostly present in the marine environment. Bacteriovoracia were increasingly abundant in deeper ocean, while Bdello-group2 prefers to inhabit the epipelagic zone. Comparative genomics demonstrated that marine Bdellovibrionota species evolved to have genes involved in osmolyte metabolism. Oligoflexia may be a potential biocontrol agent of pathogens beyond bacteria since we discovered genes encoding chitin-binding protein and chitinase.

## 2. Methods

### 2.1. Genome Collection from Public Databases and Quality Control

We recruited 148 Bdellovibrionota genomes from the GTDB database [8] (data collected until July 2020). Eight genomes for model Bdellovibrionota were downloaded from the national center for biotechnology information (NCBI). All the 156 genomes (Table S1) were reclassified with GTDB-tk (v.1.0.1) [8]. Quality control of all the genomes was conducted using CheckM (v.1.1.2) [19]. Medium- and high-quality genomes (Table S2) used for further analysis were selected with the following standards: (1) completeness score was more than 70%; (2) contamination rate was less than 10%; (3) the number of conserved proteins derived from CheckM program was at least 22. The sources of the genomes and metagenomes were obtained from the NCBI Biosample and Bioproject.

### 2.2. Marine Water Samples Collection, DNA Extraction and Sequencing

About 20 L marine water samples were in situ filtered from the Mariana Trench during *R/V* TS09 by an in situ microbial filtration and fixation (ISMIFF) apparatus and sampling procedure had been described in our previous report [20]. 20L water samples were also collected at different depths by Niskin bottles from the South China Sea during an *R/V* Tan Kah Kee cruise and *R/V* Haigong623 equipped with the remotely operated vehicle (ROV) Haixing6000 (Table 1). We used 0.22 μm polycarbonate membranes (Merck Millipore, Bedford, MA, USA) for microbial filtration and were then frozen at −80 °C degree immediately until use. The polycarbonate membranes were cut into small pieces for DNA extraction using MO BIO Power Soil DNA Isolation Kit (MoBio, Carlsbad, CA, USA) according to the manufacturer’s instruction. The quality and quantity of the DNA extraction were evaluated by 1% agarose gel electrophoresis and Qubit 3.10 Fluorometer (Invitrogen, Life, Carlsbad, CA, USA). The good-quality DNA was first sheared randomly to fragments of around 500 bp by a focused ultrasonicattor (Covaris M220) and used for metagenomic library preparation with TruSeq^®^ Nano DNA LT Sample Prep Kit (Illumina, San Diego, CA, USA). The high-throughput sequencing was performed on an Illumina Miseq platform (2 × 300 bp).

### 2.3. Raw Data Processing, de Novo Assembly and Metagenomes Binning

The quality of metagenome raw data was assessed by FastQC (v.0.11.8) [21] . After Fastp (v0.20.0) [22] treatment, repeated sequences produced by the sequencing platform were removed by Fastuniq [23] and then the clean reads were assembled with MEGAHIT (v.1.2.5-beta) [24]. The contigs >2000 bp were selected for genome binning with MetaWRAP (v1.2) (models including metabat2, maxbin2 and concoct) [25]. The MAGs were then taxonomically classified by GTDB-tk classifier [8] and five Bdellovibrionota MAGs were selected by the cut-off described above (>70% completeness and <10% contamination).

### 2.4. Calculation of Bdellovibrionota Relative Abundance in Deep Sea

We extracted 16S rRNA sequences of Bdellovibrionota genomes and MAGs by using rRNA_hmm_fs_wst (v.0) [26] and used to create a 16S rRNA database. The Bdellovibrionota 16S rRNA metagenomic Illumina tags (miTags) in Tara Ocean [27] and Mariana marine water metagenomes [28,29] (Table S3) were identified by BLASTN [30] (v.2.9.0) (E-value, 1 × 10^−5^) against the 16S rRNA sequence database of Bdellovibrionota. Only the 16S miTags longer than 100 bp and >97% identity to a reference in the database were selected for further calculation of their relative abundance in the marine samples. The Tara Ocean data were downloaded (http://ocean-microbiome.embl.de/data/miTAG.taxonomic.profiles.release.tsv.gz accessed on 22 February 2021). A *t*-test was performed for significance analysis.

### 2.5. Genome Annotation and Metabolic Reconstruction

Open reading frames (ORFs) were predicted by Prodigal (v.2.6.3) [31] and were then annotated by Kofamscan [32] (v.1.0.0; -f mapper) against the Kyoto Encyclopedia of Genes and Genomes (KEGG) database [33] (Release 92) and by BLASTP (v.2.9.0) [34] against Cluster of Orthologous Groups of proteins (COG) database (COG_2019_v11.0) [35]. Carbohydrate related enzymes were searched with hmmscan (v. 3.2.1) [36] against the Carbohydrate Active EnZyme (CAZy) database (dbCAN-HMMdb-V7) [37]. Peptidases were identified by BLASTP against the MEROPS database [38] (pepunit.lib; -id 50; -e 1e^−10^). Biosynthetic gene clusters of Bdellovibrionota were identified by using antiSMASH (v.4.2.0) [39] using default parameters and further confirmed BLASTP against MIBIG data (v.2.0) [40] as described elsewhere [41]. A Wilcoxon test in R package was conducted to identify the genes significantly different in abundance between different groups.

### 2.6. Phylogenetic Analysis of Conserved Proteins and 16S rRNA Genes

Average nucleotide identity (ANI) analysis was executed by the pyani (v.0.2.9) (software available from https://github.com/widdowquinn/pyani/releases; released on 21 May 2019) to remove redundant genomes with ≥98.5% identity [42]. A total of 86 non-redundant Bdellovibrionota genomes were used for phylogenetic analysis (Table S2). The conserved proteins of genomes were aligned using hmmerAlign.py (called by CheckM). The alignment of 43 concatenated conserved proteins (Table S2) was used for reconstruction of phylogenomic tree with RAxML [43] (v.8.2.12; -# 1000; -m PROTGAMMALG) after optimization with trimAl [44] (v.1.4). All the 16S rRNA sequences extracted by rRNA_hmm [26] were treated with CD-HIT [45] (v.4.8.1; -c 0.97; -n 10; -G 1; -M 10000) to remove the same species. Two 16S rRNA sequences of *E. coli* were added as an out-group. All 16S rRNA sequences were aligned with MAFFT [46] (v.7.407) and optimized with trimAl. RAxML (v. 8.2.12; GTRCAT model) was used to build a 16S rRNA phylogenetic tree with bootstrap values based on 1000 replicates. All phylogenetic trees were visualized with iTOL [47].

### 2.7. Availability of Data and Materials

The genomes from public databases were listed in Table S1 and the MAGs assembled in this study can be obtained from NCBI Bioproject (PRJNA668648).

## 3. Results

### 3.1. Phylogenomics of Bdellovibrionota

We recruited 156 genomes of Bdellovibrionota from the GTDB and NCBI databases. Five MAGs that represent deep-sea Bdellovibrionota were retrieved from marine water samples of the Mariana Trench, the Bashi Strait and the South China Sea with depths ranging from 1646 m to 5992 m (Table 1). The five deep-sea MAGs were in the size range of 2.19~5.88 Mbp with more than 70% completeness and less than 3% contamination (Table 1).

**Figure 1:**
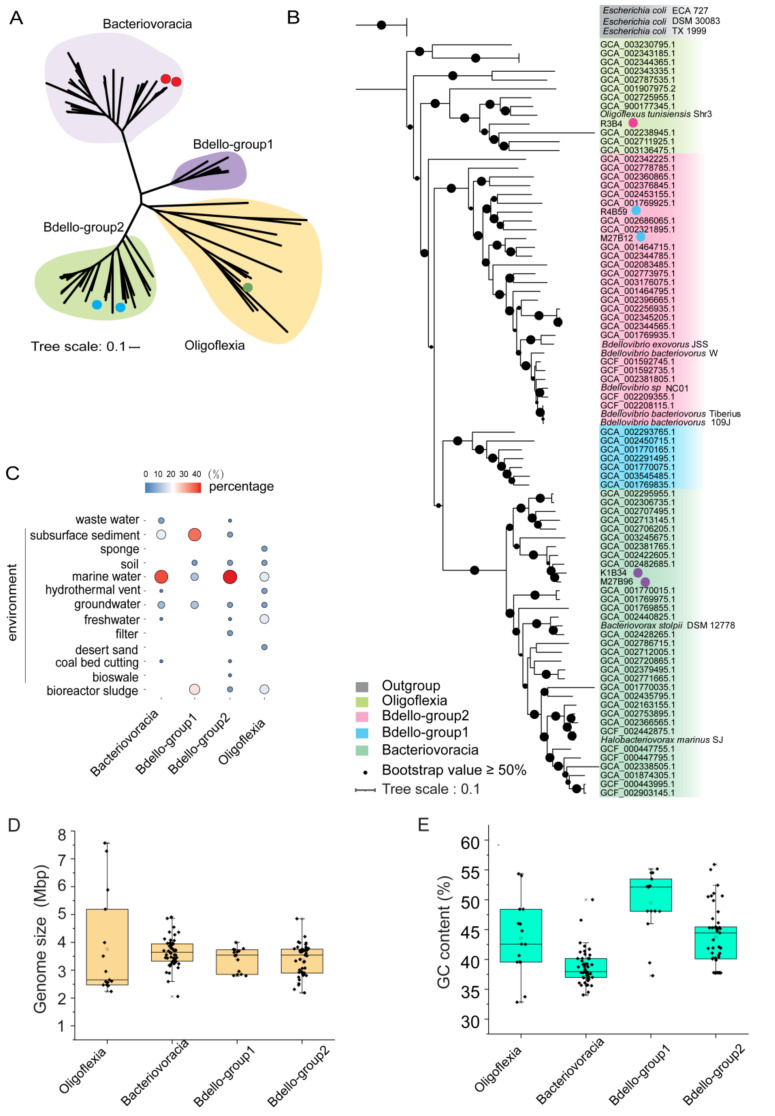
Phylogenomic analysis, distribution and genomic features of Bdellovibrionota. (**A**) Unrooted maximum-likelihood (ML) phylogenomic tree of Bdellovibrionota. The ML tree was constructed based on 43 concatenated conserved proteins with the PROTGAMMALG model. The MAGs from this study were marked with a dot. (**B**) Rooted ML phylogenomic tree of Bdellovibrionota. The tree was built up using three *E. coli* genomes as an outgroup. The genomes with a dot were marine MAGs from this study and the references were shown as their Genome Taxonomy Database (GTDB) accession numbers or species names. (**C**) Environmental source of Bdellovibrionota genomes. The size and color of dots represent the percentage of the sources in each group. (**D**) genome size and (**E**) cytosine and guanine (GC) content of Bdellovibrionota genomes were also plotted.

Construction of a phylogenomic tree using 43 conserved proteins of 86 non-redundant medium- and high-quality genomes (Table S2) displayed four phylogenetic groups (subclades), which were then named as ‘Bacteriovoracia’, ‘Oligoflexia’, ‘Bdello-group1’ and ‘Bdello-group2’ (Figure 1A,B). The phylogenetic relationships between the groups were consistent with those based on 16S rRNA genes (Figure S1). The five deep-sea MAGs were distributed into three Bdellovibrionota groups except for the Bdello-group1 (Figure 1A,B). The available isolation sources of the samples and genomes were collected and summarized. These genomes were mainly obtained from marine water, subsurface sediment and bioreactor sludge, waste water and ground water (Figure 1C). Bdello-group2 and Oligoflexia were found in more diversified environments (Figure 1C). Marine and ground water were the most common sources for Bdellovibrionota, which coincides with their preference for an environment with low viscosity [48]. About 43.40% of Bacteriovoracia and 46.81% of Bdello-group2 genomes were derived from marine waters (Figure 1C), perhaps implying their important role in oceans. The wide spread of Bdellovibrionota may be attributable to a two-phase lifestyle and quick response to the transformation between the phases [49].

Among the four groups, the size of Oligoflexia genomes varies considerably (Figure 1D). This is true for their genomic GC contents ranging from 32.83% to 54.35% (Figure 1E), which might be stemmed from the presence of many copies of transposase genes in their genomes (Figure S2). The average GC content of Bdello-group1 genomes was highest with a mean value more than 50% (Figure 1E). Note that about 30% of the Bdello-group1 genomes were obtained from sludge bioreactors (Figure 1C).

### 3.2. Relative Abundance of Bdellovibrionota in Marine Water Zones

**Figure 2:**
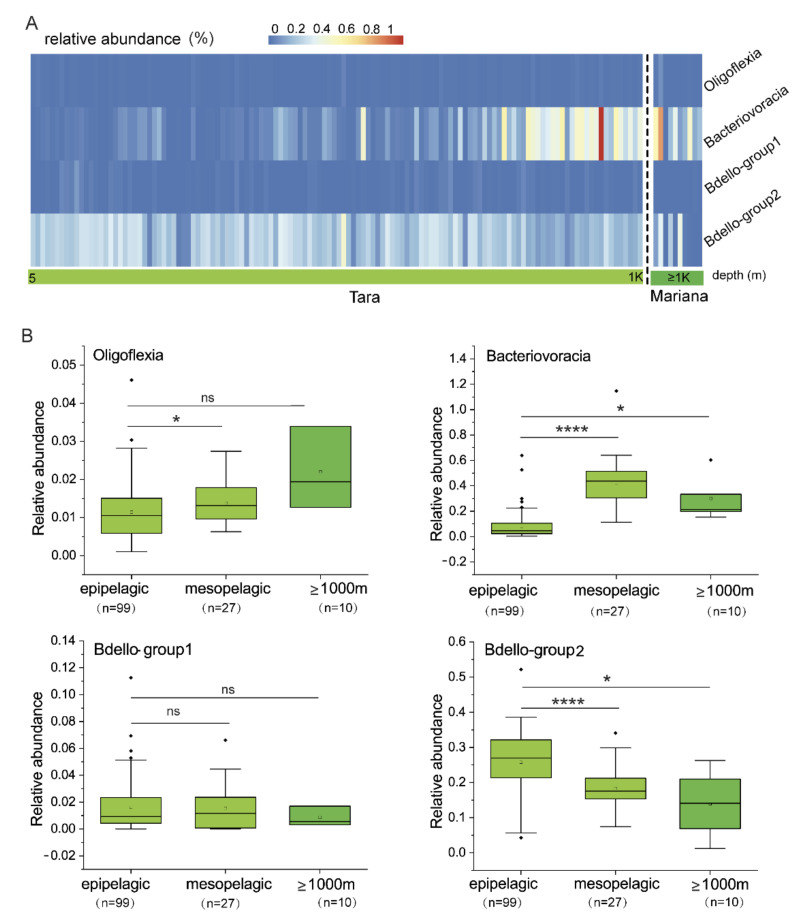
Vertical distribution of Bdellovibrionota in marine waters. (**A**) Relative abundance of Bdellovibrionota was calculated as percentage of Bdellovibrionota 16S miTags in metagenomes of the Tara Ocean project (depth from 5 m to 1000 m) and the Mariana Trench (≥1000 m) (Table S3) (Gao et al., 2019; Li et al., 2019). (**B**) Relative abundances of Bdellovibrionota groups in different marine layers were plotted. A *t*-test was performed to compare the relative abundances at different layers. (****, *p* < 0.0001; *, *p* < 0.05).

To examine vertical distribution of the four Bdellovibrionota groups (Figure 1A) in marine water zones, the 16S miTags of the Tara Ocean [27] and Mariana marine water metagenomes [28,29] were used to calculate relative abundance of Bdellovibrionota. Bacteriovoracia and Bdello-group2 were relatively abundant in oceans, compared to the other two groups (Figure 2A). The relative abundance of Bacteriovoracia increased with water depth, whereas a reverse trend was observed for Bdello-group2. Statistical analysis showed that epipelagic layer had significantly more abundant Bdello-group2 than the other deep-sea layers, whereas significantly more abundant Bacteriovoracia were distributed in deeper layers (*t*-test, *p* < 0.05) (Figure 2B). These results strongly indicate that Bacteriovoracia was relatively more prevalent in the deeper marine environment. Conversely, Bdello-group2 prefers to inhabit the epipelagic zone. Oligoflexia and Bdello-group1 are associated with very low relative abundance in marine environment (Figure 2B). Oligoflexia showed increased tendency with significance between epipelagic and mesopelagic zones (*t*-test, *p* < 0.05), whereas there was no significance when comparing the epipelagic and deeper layer (≥1000 m) (Figure 2B). Those results showed that vertical distribution pattern of Bdellovibrionota differed between the groups, suggesting considerable differences in their gene profiles.

### 3.3. Metabolic Potentials (Reconstruction) of Bdellovibrionota

**Figure 3:**
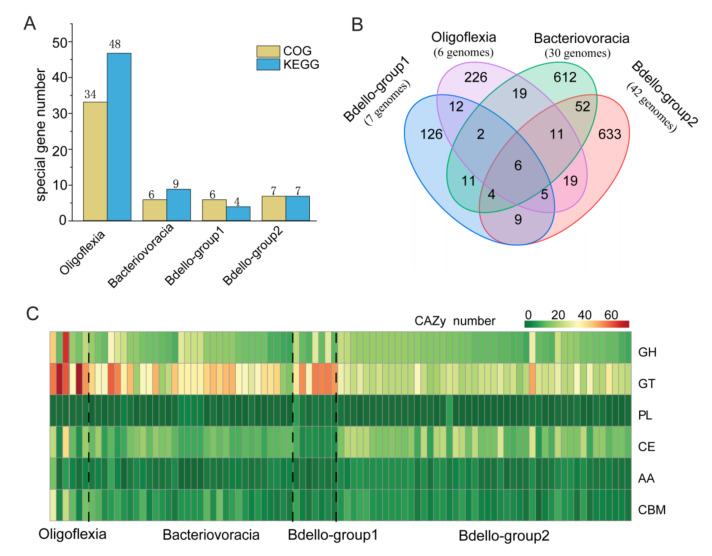
Gene content difference among Bdellovibrionota groups. (**A**) Number of group-specific Cluster of Orthologous Groups of proteins (COG) and Kyoto Encyclopedia of Genes and Genomes (KEGG) genes. (**B**) Venn diagram showing group-specific and shared peptidases of Bdellovibrionota. (**C**) Heatmap illustrating differences in number of carbohydrate active enzyme (CAZy) classes. GH, glycoside hydrolases; PL, polysaccharide lyases; GT, glycosyl transferases; CE, carbohydrate esterases; CBM, carbohydrate-binding modules; AA, auxiliary activities.

The 80 high-quality (>90% completeness and <5% contaminant) genomes from public databases and five deep-sea MAGs binned for this study were used for gene annotation against KEGG [33], COG [35], MEROPS [38] and CAZy databases [37]. Discrepancies of the Bdellovibrionota groups in metabolism and environmental adaptation were detected by comparing the annotation results. When group-specific genes (only present in more than 50% genomes of one group) were selected from KEGG and COG annotation results, we found that Oligoflexia genomes contained 48 KEGG genes and 34 COGs as group-specific genes, remarkably more than other groups (Figure 3A). The peptidase-coding genes found in MEROPS annotation were compared, which showed that six peptidase genes were shared by four groups and each group also contained unique peptidases (Figure 3B). Oligoflexia differs from other groups of Bdellovibrionota with higher average number of unique peptidases (Figure 3B), which indicated that Oligoflexia may degrade a wide spectrum of peptides for survival or adaptation [50]. Based on CAZy annotation, Oligoflexia genomes encode more enzymes of glycosyl transferase (GT) families than other groups (Figure 3C). GT enzymes can catalyze the transfer of sugar moieties onto aglycons, which is related to transformation of many natural products [51]. This indicates that Oligoflexia may heavily depend on degradation of carbohydrate from prey bacterial cells and environment. In addition, the secondary metabolite gene clusters encoding non-ribosomal peptide synthetases (NRP) and for synthesis of NRP-polyketide, polyketide, polyketide-saccharide, ribosomally synthesized and post-translationally modified peptides (RiPP) and saccharide were present in one or more genomes of each group (Table S4). The results might indicate that Bdellovibrionota are potentially capable of producing secondary metabolite.

**Figure 4:**
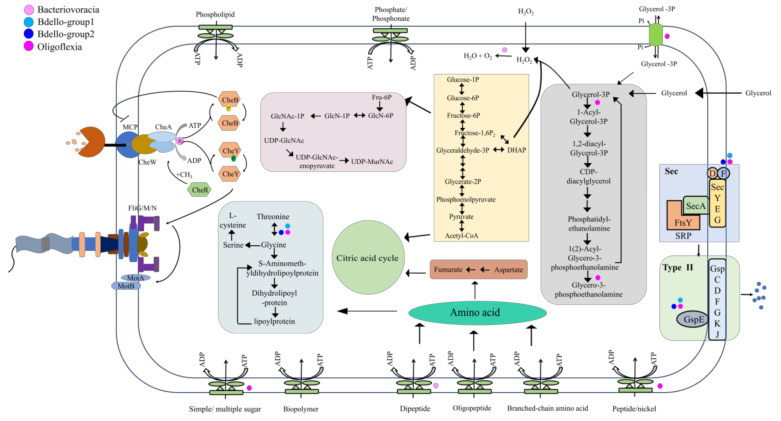
Metabolism reconstruction of Bdellovibrionota. The schematic metabolism and structural components of Bdellovibrionota were predicted based on KEGG annotation results for the four groups. The genes responsible for the steps without a dot are identified in all the four groups of Bdellovibrionota; if not, dots were used to denote their presence in group(s).

To disentangle the metabolic and adaptive capacities of Bdellovibrionota, metabolism reconstruction was carried out and presented (Figure 4). If at least 50% genomes of a group harbor a gene, the group was regarded to have the gene. As bacterial predators, Bdellovibrionota genomes all harbor genes that encode an almost full set of subunits involved in mobility (FlgKGI, FliG/M/N and MotA), chemotaxis (CheY, B, W, A, R and monocyte chemoattractant protein, MCP), type II secretion system and some ABC transporters (Table S5). These chemotactic proteins may work effectively and help Bdellovibrionota to sense abundance of prey bacteria and toxic chemicals [52]. Multicopies of *mcp* (up to 27 copies) and *motA* genes were predicted in an Oligoflexia genome (GCA_001907975.2 or [32] in this study) (Table S6). Artificial mutations of *motA* affected integrality of flagellar [53], which indicated that MotA is important in flagellar assembly. MCP, a methyl accepting chemotaxis protein, was reported to act as an enhancer for predatory ability of Bdellovibrionota [54]. In addition, *cheB* was predicted in all the groups (71.42% of Oligoflexia genomes; all of the Bacteriovoracia genomes; 57.14% of Bdello-group1 genomes and 89.13% of Bdello-group2 genomes) (Table S5). During bacterial rapid adaptation and responding to the local gradients of attractants or repellents, protein CheB, a methylesterase, functions in covalent modification of membrane receptors [55]. A gene encoding a methyltransferase CheR is present in all the Bdellovibrionota groups (Table S5) [56]. Methylation enhances clockwise signals of the motors, while demethylation attenuates them by means of a feedback loop that is managed by CheB and CheR with respect to methylation rate of receptors [57]. This is the nature of motor transition between clockwise and counterclockwise to respond to the changes of environmental factors. Bdellovibrionota may hunt other bacteria with assistance of methylated modification related chemotaxis proteins (CheB, CheR and MCP) for precise control of bacterial motility.

After invasion into periplasmatic space of prey bacteria, Bdellovibrionota bacteria begin to degrade the cytoplasmic contents of the prey cell [58]. Amino acids, glycerol and lipid of cellular components are all nutrients for Bdellovibrionota [58]. Glycerol, a simple organic molecule, can be produced by several metabolism pathways (such as glycolysis and lipid catabolism). The coding genes of glycerol kinase (GK) and glycerol-3-phospahte-dehydrogenase (GLPD) were predicted in almost all Bdellovibrionota genomes (Table S6), which indicates that Bdellovibrionota can utilize glycerol for glycolysis (Figure 4) as reported previously [58]. The by-product of glycerol oxidation is H_2_O_2_, which is cytotoxic [59] and can be used to destroy cell wall structure of prey bacteria after free diffusion of H_2_O_2_ into the prey periplasmic space. The coding gene of sn-glycerol-3-phosphate acyltransferase (PlsB), the first enzyme for membrane phospholipid biosynthesis [60], was predicted only in Oligoflexia genomes (Table S6). This suggests that glycerol may be used for membrane phospholipid biosynthesis in rapid growth phase of Oligoflexia and thus the glycerol metabolism was diversified in Bdellovibrionota.

The metabolism reconstruction shows that there were numerous kinds of ABC transporters for uptake of phospholipid, lipoprotein, oligopeptide and branched-chain amino acids (Figure 4). They were also useful to import nutrients from environment in free-living phase. The strategies might help Bdellovibrionota to adapt to oligotrophic, low temperature and hydrostatic pressure in deep sea zones.

The degradation and assimilation of nutrients are essential processes during the predatory growth phase of Bdellovibrionota. When Bdellovibrionota invade the prey bacteria and entered into growth phase, the percentage of transcribed genes increased from 33% to 85% [49]. In Bdellovibrionota, amino acids derived from prey bacterial cell, environmental source and oligopeptide digestion were fed in different metabolism pathways [58]. For example, aspartate is linked with citric acid cycle and glycine may involve into lipoylprotein metabolism (Figure 4). During the release stage of Bdellovibrionota progeny, a large amount of outer cellular components was required such as peptidoglycan that is the basic unit of the cell wall [61]. Bdellovibrionota might use fructose 6-phosphate for producing uridine diphosphate (UDP)-N-acetylmuramic acid (UDP-MurNAc) (Figure 4) and subsequent biosynthesis of peptidoglycan. The Sec-type II system is almost complete in all Bdellovibrionota groups (Figure 4 and Table S5). The Type II secretion system can exude numerous bacterial toxic proteins and lytic enzymes such as proteases and lipases, which is important for bacteria to digest nutrients in the prey cell and environment [62]. Bdellovibrionota may heavily depend on their type II secretion system in the whole lifecycle. During the attack phase, the secreting proteins can probably be used to catch prey bacteria and partly destruct the outer membrane structure for entry; during the predatory growth phase, they are likely useful for cellular component digestion and complete prey cell lysis.

### 3.4. Genes Involved in Survival of Bdellovibrionota in the Marine Environment

Although many Bdellovibrionota genomes were obtained from the marine environment, there is not a special group exclusively consisting of marine species (Figure 1). Given the characteristics with high hydrostatic pressure, oligotrophy and low temperature in the deep sea, there must be some genes in Bdellovibrionota for deep-sea adaptation. To reveal these genes, we divided each group of the Bdellovibrionota genomes into two parts, non-marine and marine, according to their sample sources. The marine genomes of Bdellovibrionota mostly belonged to Bacteriovoracia and Bdello-group2 (Figure 1C). A comparison between the two groups showed that the marine genomes have *betT*/*betS* genes (77.78% of Bacteriovoracia marine genomes and 57.89% of Bdello-group2 marine genomes) that take part in betaine biosynthesis via the choline pathway because of their high-affinity to choline (Table 2). Betaine is one of known major organic molecules that protect bacteria from damage of hypersaline/high hydrostatic pressure by stabilizing the natural conformation of proteins and preventing their aggregation [63]. Although it is reported that choline and glycine betaine are nearly ubiquitous in bacteria [63], the percentage of *betT*/*betS* in the marine group is much higher than that in non-marine group (77.78% vs. 21.05% of Bacteriovoracia and 57.89% vs. 0 of Bdello-group2) in this study (Table 2). This indicates that *betT*/*betS* genes in the marine Bdellovibrionota may be critical in protecting them from the high hydrostatic pressure by importing choline/glycine/proline betaine from the sea water or the prey bacterial cells. Apart from *betT*/*betS*, the gene named *mscS* (Table 2), contributing to normal resistance to hypoosmotic shock, was predicted in 89.47% of Bdello-group2 marine genomes. The MscS channel is integrated in the cell membrane, which prevents burst of the cell in hypotonic environments [64,65]. It may work with BetT/BetS in a feedback model for maintaining and rescuing Bdellovibrionota (Bdello-group2) under the various marine chemical gradients.

The special physicochemical parameters of the ocean lead to diverse resistance mechanisms of marine microbes, such as detoxification. The H_2_O_2_ degrading genes were predicted in Bacteriovoracia marine genomes, and were perhaps involved in prevention against self-toxification of H_2_O_2_ induced by the byproduct of glycerol oxidation. The genes *osmC* (77.78%), *catA* (55.56%) and *katE* (55.56%) were present in Bacteriovoracia marine genomes (Table 2). These genes are probably related to protect cells from the toxic effects of H_2_O_2_ that acts as a bactericide [66]. OsmC is a protein induced by osmotic pressure [67]. Interestingly, its malfunction reduced fitness and elevated sensitivity to oxidative stress of *E. coli* [68]. Therefore, OsmC may participate in defense against high salinity and H_2_O_2_ (a defensive molecule that may also be produced by prey bacteria) in Bacteriovoracia (Figure 4). As for *catA*, it was up-regulated in *Streptomyces coelicolor* treated with H_2_O_2_ during no-stationary phase and it was required for *Streptomyces coelicolor* growth under aerobic condition [69,70]. The function of *katE* has not yet been determined, but it is predicted to regulate the concentration of H_2_O_2_ [71,72]. H_2_O_2_ may be scavenged and degraded in Bacteriovoracia with the co-operation of OsmC, CatA and KatE for survival in deeper ocean (Figure 2 and Table 2).

In Bdello-group2 marine genomes, there are some genes involved in cell wall degradation, recycling, organization and biogenesis (Table 2). Penicillin-binding proteins are important for Bdellovibrionota to break up prey cell wall [73]. MltA encoded by 57.89% of Bdello-group2 marine genomes is a murein degrading enzyme that is located in outer-membrane and can interact with penicillin-binding protein 2 [74,75], which is important for marine Bdello-group2 as the predator of other Gram-negative bacteria. The *MreD* gene was present in 57.89% of Bdello-group2 marine genomes (Table 2) and is essential for lateral peptidoglycan synthesis [76]. We identified *anmK* gene in 52.63% of Bdello-group2 marine genomes (Table 2). The enzyme encoded by *anmK* plays a role in cell wall recycling though transforming 1,6-anhydro-N-acetylmuramic acid (anhMurNAc) to N-acetylglucosamine-phosphate (GlcNAc-P) [77]. Usually, the source of anhMurNAc is derived from its own cell wall murein [58] but, as predators, marine Bdello-group2 may reuse the component of prey bacteria for cell wall formation of the next generation. ECM4, one member of glutathione transferase that might be related to protect cell such as involved in resisting the toxicity of quinone [78], found in 52.63% of Bdello-group2 marine genomes (Table 2) was proposed as a protein involved in cell wall biogenesis (integrity) [79]. Under deep-sea oligotrophic condition, these genes may all be associated with the whole process from murein-degrading, anhMurNAc recycling and cell wall biogenesis for proliferation of Bdello-group2 marine bacteria.

There are two genomes of Bdello-group1 (GCA_002722705.1 and GCA_002450715.1) and only one genome of Oligoflexia (R3B4) from marine. The discrepancies in genes between marine and non-marine groups were also demonstrated (Table 2). The genes involved in synthesis of osmoprotectants were also predicted in marine Bdello-group1 and Oligoflexia genomes, such as *betT* and *mscS* in Bdello-group1 and *cmo* in Oligoflexia (Table 2). CMO (choline monooxygenase) can catalyze the first step of glycine betaine synthesis [80], which indicates that glycine betaine can be used as an osmoprotectant in marine Oligoflexia as well. In addition, there are many genes involved in transport or transformation of sugar as osmoprotectant, such as *malK*, *msmX*, *rgpF* and *algI* genes in Oligoflexia (Table 2) [81,82,83,84]. In light of all these results, betaine as an osmoprotectant is commonly used among different groups of Bdellovibrionota, all having a variety of genes for fitness in the ocean.

### 3.5. Oligoflexia as Potential Defender against Eukaryotic Pathogens

**Figure 5:**
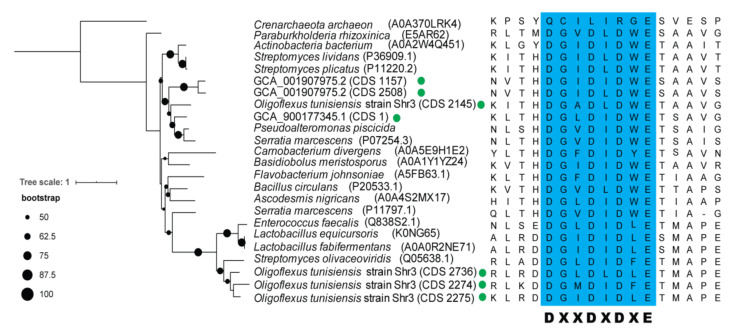
Phylogenetic analysis and conserved motif of chitinases from Oligoflexia. The phylogenetic tree of chitinases was built with RAxML (PROTGAMMALG model). The bootstrap values based on 1000 replicates were denoted by the size of the black dots on the branches. The Oligoflexia genomes are marked with a green dot. The conserved motif of chitinases is highlighted with blue background in the multiple sequence alignment.

In light of the comparison among Bdellovibrionota groups, Oligoflexia seems to be special in gene content. The chitinase gene (K01183) has many copies in Oligoflexia genomes (Figure S2), but none in other Bdellovibrionota groups. The presence of genes encoding chitinase and chitin-binding protein (Figure S2) indicates that some Oligoflexia may be able to degrade chitin [85,86]. The phylogenetic tree of the seven chitinases extracted from the Oligoflexia genomes and other species supports their close relationships with known homologs (Figure 5). To further verify the predicted ORF of the chitinases, multiple alignment was performed to validate the conserved motif ‘DXXDXDXE’ of chitinases (Figure 5) [87]. In addition, the Oligoflexia genomes contained a gene coding for chitin-binding protein (Figure S3 and Table S7). In addition, sec signal peptide (Sec/SPI) and lipoprotein signal peptide (Sec/SPII) were detected in the ORF of chitinase (Table S7). These results imply that some of Oligoflexia can utilize chitin in the environment as nutrient and/or attack preys with chitin ultracellular structure. In this study, the chitinase and chitin-binding protein coding genes were focused for the first time in Oligoflexia. Pathogenic fungi have chitin as a main cell wall component [88], which allows us to speculate that Oligoflexia may be used to defend against eukaryotic pathogens aside from bacterial pathogens.

## 4. Discussion

In this study, the phylogenomics of Bdellovibrionota inhabiting different niches revealed that the genomes were scattered into four groups without niche-specific groups (Figure 1A–C and Figure S1). The universal distribution of Bdellovibrionota may be due to their quick transformation in response to environmental changes [49]. Our comparison of genomic traits allows for a glance of their differences in peptidases, CAZymes, and metabolic spectrum (Figure 3B,C and Figure 4). The wide range of genome size and GC content of Bdello-group1 genomes is likely ascribed to frequent contacts with the diverse microbes in bioreactor sludges. In this study, the relative abundances of four groups of Bdellovibrionota in vertical marine water zones indicate preference of Bdellovibrionota groups to different depths. These observed distinctions may be related to the biotic (gene profiles of Bdellovibrionota, prey cell abundance and other bacterial predators) or abiotic (nutrients, temperature and hydrostatic pressure) factors in the marine environment. All the factors and predatory role of Bdellovibrionota in the control of microbial diversity need to be studied further.

Our predicted functions of the proteins responsible for the flagellar assembling, chemotaxis, and type II secretion system agree with the predatory lifestyle of Bdellovibrionota. Moreover, penicillin-binding proteins were probably important for Bdellovibrionota, because it may help Bdellovibrionota destroy the prey cell wall and invade prey cell periplasm. In addition, because of oligotrophic, low temperature and hydrostatic pressure in deep sea zones, marine Bdellovibrionota probably adapt for survival with osmoprotectant uptake, H_2_O_2_ detoxification and recycling of cell wall components. In particular, the potential capacity to reuse the cell wall and to lysis prey cell during release of Bdellovibrionota progeny is of importance to promote the circulation of geochemical elements in the deep ocean. Previous studies revealed that viral diversity at different scales affected the microbial communities in deep ocean [89]. Bacterial predators, such as Bdellovibrionota, may also influence the population size of their preys and make the microbial community more diversified in different niches as previously reported [90]. In addition, infection by MDR bacteria could be controlled with assistance of Bdellovibrionota [6]. Our result suggests that Oligoflexia seem to be potential new candidates for controlling eukaryotic pathogens, with respect to the finding of chitinase and chitin-binding protein coding genes. In addition, the presence of genes for biosynthesis of secondary molecules indicates that Oligoflexia might be candidate producers of novel biosynthetic compounds.

## 5. Conclusions

In this study, Bdellovibrionota was revealed to have four phylogenetic groups, all containing the genes in most of their genomes for two-phase predatory lifestyle. Their vertical distribution in the ocean was exhibited. Bacteriovoracia was more prevalent in the deep-sea zones. Genomics traits demonstrated that marine Bdellovibrionota were able to metabolize osmoprotectant and degrade cell wall. The chitinase and chitin-binding protein encoding genes were for the first time focused upon in Oligoflexia. However, the function and regulation of these genes involved in pathogenic prevention need to be verified by experiments.

## Figures and Tables

**Table 1:** Information of metagenome-assembled genomes (MAGs) of marine Bdellovibrionota.

Bin_id	K1B34	M27B12	M27B96	R3B4	R4B59
Depth (m)	1000	3000~5000	3000~5000	1646	5992
Sampling site	116.47°	142.01°	142.01°	120.03°	123.31°
(E; N)	18.01°	11.16°	11.16°	21.87°	22.82°
Com. (%)	75.16	98.21	80.15	81.83	71.94
Con. (%)	0.00	0.89	2.98	2.52	0.89
N50 (bp)	6396	295,316	5991	7831	3968
No. contigs	597	24	684	923	570
Genome size (bp)	3,229,602	4,849,992	3,554,382	5,883,831	2,188,444
No. CDSs	3741	4431	4172	5667	2566

R and K in Bin_id represent the South China Sea; M denotes the Mariana Trench. Com., completeness; Con., contamination; N50, the shortest contig length required to cover 50% of the genome. CDS, coding sequence.

**Table 2:** Candidate genes for adaptation to the marine environment.

Genes Enriched in Marine Genomes of Bdellovibrionota.Group	KEGG	Non-Marine	Marine	Function
Bdello-group2	K03442	11%	89%	*mscS*; small conductance mechanosensitive channel
	K08304	6%	58%	*mltA*; membrane-bound lytic murein transglycosylase A [EC:4.2.2.-]
	K03571	17%	58%	*mreD*; rod shape-determining protein MreD
	K02168	0%	58%	*betT, betS*; choline/glycine/proline betaine transport protein
	K00147	11%	53%	*proA*; glutamate-5-semialdehyde dehydrogenase [EC:1.2.1.41]
	K07393	0%	53%	*ECM4, yqjG*; glutathionyl-hydroquinone reductase [EC:1.8.5.7]
	K09001	11%	53%	*anmK*; anhydro-N-acetylmuramic acid kinase [EC:2.7.1.170]
Bdello-group1	K08641	0%	100%	*vanX*; zinc D-Ala-D-Ala dipeptidase [EC:3.4.13.22]
	K01273	0%	100%	*DPEP*; membrane dipeptidase [EC:3.4.13.19]
	K05995	0%	100%	*pepE*; dipeptidase E [EC:3.4.13.21]
	K15773	0%	100%	*hipB*; HTH-type transcriptional regulator/antitoxin HipB
	K03442	0%	100%	*mscS*; small conductance mechanosensitive channel
	K02168	0%	100%	*betT, betS*; choline/glycine/proline betaine transport protein
	K06218	0%	100%	*relE, stbE*; mRNA interferase RelE/StbE
	K07339	17%	100%	*hicA*; mRNA interferase HicA [EC:3.1.-.-]
	K18843	0%	100%	*hicB*; antitoxin HicB
	K19092	0%	100%	parE1_3_4; toxin ParE1/3/4
Bacteriovoracia	K02168	21%	78%	*betT, betS*; choline/glycine/proline betaine transport protein
	K04063	11%	78%	*osmC*; lipoyl-dependent peroxidase
	K19271	5%	56%	*catA*; chloramphenicol O-acetyltransferase type A [EC:2.3.1.28]
	K03781	0%	56%	*katE, CAT, catB, srpA*; catalase [EC:1.11.1.6]
Oligoflexia	K01177	0%	100%	beta-amylase [EC:3.2.1.2]
	K10111	0%	100%	*malK*; multiple sugar transport system ATP-binding protein [EC:3.6.3.-]
	K10112	0%	100%	*msmX*; multiple sugar transport system ATP-binding protein
	K07272	0%	100%	*rgpF*; rhamnosyltransferase [EC:2.4.1.-]
	K05841	0%	100%	sterol 3beta-glucosyltransferase [EC:2.4.1.173]
	K03313	0%	100%	*nhaA*; Na+:H+ antiporter, NhaA family
	K19294	0%	100%	*algI*; alginate O-acetyltransferase complex protein AlgI
	K00499	0%	100%	*CMO*; choline monooxygenase [EC:1.14.15.7]

## Data Availability

The data presented in this study are available on request from the corresponding author.

## Supplementary Materials

The following are available online at https://www.mdpi.com/article/10.3390/microorganisms9040757/s1, Figure S1: Rooted phylogenetic tree of Bdellovibrionota 16S rRNA genes. Figure S2: Copy number of interested KEGG genes of Oligoflexia from different environments. Figure S3: Phylogenetic tree of chitin-binding proteins encoded by Oligoflexia genomes. Table S1: GTDB taxonomy of Bdellovibrionota genomes and MAGs from public databases. Table S2: Medium- and high-quality Bdellovibrionota genomes and MAGs. Table S3: Information of deep-sea water metagenomes. Table S4: Secondary metabolite gene clusters of Bdellovibrionota. Table S5: Genes related to lifestyle of Bdellovibrionota as predator. Table S6: KEGG annotation results. Table S7: BLASTP results of chitinase and chitin-binding protein.
