# Supplementary material for: Phylogenomic Insights into Distribution and Adaptation of Bdellovibrionota in Marine Waters
Source: Microorganisms. 2021 Apr 3;9(4):757. doi: 10.3390/microorganisms9040757 (PMC8067016; doi:10.3390/microorganisms9040757)
Supplement: Supplementary file 1 [file microorganisms-09-00757-s001.zip › Figures_supp.doc]

**Figure S1. Rooted phylogenetic tree of Bdellovibrionota 16S rRNA genes.**

Maximum-likelihood (ML) phylogenetic tree of 16S rRNA genes extracted from high-quality genomes of Bdellovibrionota were reconstructed using PROTGAMMALG model. Blue dots refer to the MAGs retrieved from marine waters of this study.

**Figure S2. Copy number of interested KEGG genes of Oligoflexia from different environments.**

The special genes are only present in Oligoflexia genomes inhabiting different environments. Chitinase (more than one copy) and chitin-binding protein coding genes were predicted in some genomes. The accession numbers of the genomes can be found in Table S1. The isolation sources of the genomes are listed as following: Shr3 (desert); ref130 (not available); ref32, ref49 and ref48 (freshwater); R3B4 (deep-sea water).

**Figure S3. Phylogenetic tree of chitin-binding proteins encoded by Oligoflexia genomes.**

The red dots refer to amino acid sequences of chitin-binding proteins predicted based on KEGG annotation of Oligoflexia genomes.
